# The Dangers of Paternalism

**Published:** 1903-02

**Authors:** 


### Editorial.

### THE DANGERS OF PATERNALISM.

The trade journals have now become so cheap, and in some cases so well conducted, that the question naturally arises to many, Why should not the profession give up the attempt to control the publication of its records; why not allow the supply houses to assume the entire charge of the intellectual output? They are trained commercially, they know the money values of the materials with which we work, and how to put such materials upon the market. Why should they not also be able to judge of the value of our professional ideas, and know best how to put them on the market?

If this course of reasoning is allowed, we will find that a few principles which are cherished either openly or secretly by all members of the profession will have to be abandoned. In the first place, as to the idea that a liberal profession should be perfectly free and unbiassed in its investigations and in the expression of the same. How is this possible if our periodical literature is in the hands of manufacturing houses which have a commercial interest in the results of our investigations? If our work suits them, it will undoubtedly be granted full expression and illustration; but if it runs counter to their interests, business instinct and business practice will forbid its expression. Here, then, is a conflict between a professional principle and a trade principle, and the professional principle must be abandoned.

But a still greater harm comes to the profession from trade journalism by the paternalism which is thereby engendered. The trade virtually says, "My professional children, I recognize your skill in mechanical things and your advancement along intellectual lines, and I am doing my best to furnish you with suitable instruments to carry on your work. I expect as a result of your education and experience you will have ideas to express, but pray do not undertake the publication of these ideas for yourself. WE have a trade with you the profits of which will amply allow us to conduct the journals in which your thoughts can be expressed and which can be sold to you at a nominal cost. Our tempting display of instruments advertised in these journals is sufficient reward for us. WE will save you the trouble of collecting and printing your ideas; we will write editorials for you and outline the policy which you should take on important questions. By saving you the trouble of thinking, you will have so much more time to exercise your mechanical skill and make money. That is what we are both mainly striving for."

To this proposition the majority of dentists apparently agree. They have been so long accustomed to the paternal oversight of the trade that they see no objection to having their ideas collected and published for them, especially as the cost of publication is merely nominal. Such paternalism cannot contribute to the elevation and development of a profession, it is a form of bondage. Now, bondage may be so pleasant that it is not recognized as such. If it contributes to our ease and is economical we forget its repressing power. The trade would make us forget the bondage which must go with trade journalism by offering us their journals so cheaply and with so little effort to ourselves. If we accept what they so generously offer, we ought to feel bound and indebted to their commercial products. It should finally be borne in mind that at the present day the tendency of all trade is to combine and thus diminish competition and destroy individual liberty. This process has been going on for a long time among those who furnish us with supplies. It is possible that this policy may ere long be applied to trade journals, and all be eliminated save one. How short-sighted, then, to give over to foreign hands matters of such vital interest to the profession. An independent journal managed by dentists and for dentists stands as a protest to such action

